# The Influence of Mathematical Definitions on Patellar Kinematics Representations

**DOI:** 10.3390/ma14247644

**Published:** 2021-12-11

**Authors:** Adrian Sauer, Maeruan Kebbach, Allan Maas, William M. Mihalko, Thomas M. Grupp

**Affiliations:** 1 Research and Development, Aesculap AG, 78532 Tuttlingen, Germany; Allan.Maas@aesculap.de (A.M.); Thomas.Grupp@aesculap.de (T.M.G.); 2 Department of Orthopaedic and Trauma Surgery, Musculoskeletal University Center Munich (MUM), Campus Grosshadern, Ludwig Maximilians University Munich, 81377 Munich, Germany; 3 Department of Orthopaedics, Rostock University Medical Center, 18057 Rostock, Germany; Maeruan.Kebbach@med.uni-rostock.de; 4 Campbell Clinic Department of Orthopaedic Surgery and Biomedical Engineering, University of Tennessee Health Science Center, Memphis, TN 38163, USA; wmihalko@campbellclinic.com

**Keywords:** knee joint, patello-femoral joint, kinematics, cardan sequence, euler angles, conversion

## Abstract

A correlation between patellar kinematics and anterior knee pain is widely accepted. However, there is no consensus on how they are connected or what profile of patellar kinematics would minimize anterior knee pain. Nevertheless, answering this question by merging existing studies is further complicated by the variety of ways to describe patellar kinematics. Therefore, this study describes the most frequently used conventions for defining patellar kinematics, focusing on the rotations. The similarities and differences between the Cardan sequences and angles calculated by projecting axes are analyzed. Additionally, a tool is provided to enable the conversion of kinematic data between definitions in different studies. The choice of convention has a considerable impact on the absolute values and the clinical characteristics of the patello-femoral angles. In fact, the angles that result from using different mathematical conventions to describe a given patello-femoral rotation from our analyses differ up to a Root Mean Squared Error of 111.49° for patellar flexion, 55.72° for patellar spin and 35.39° for patellar tilt. To compare clinical kinematic patello-femoral results, every dataset must follow the same convention. Furthermore, researchers should be aware of the used convention’s implications to ensure reproducibility when interpreting and comparing such data.

## 1. Introduction

Patello-femoral pain has a prevalence of more than 20% in the general population [1] and a high percentage of unsatisfied patients after total knee arthroplasty complain of anterior knee pain [2,3]. Even if satisfactory patellar tracking and kinematics seem to be evident on physical exam [4,5], it is still not entirely clear, what defines good patellar tracking and how healthy patellar kinematics can be quantified [6].

Although previous studies have investigated patellar kinematics [6,7], it remains unclear what ideal patellar kinematics encompass. What we know about patellar kinematics is that the patellar native facets or prosthetic button should be centered in the trochlear groove without subluxation or tilt throughout range of motion [6,7]. Even with descriptions of how the patella tracks from full extension to flexion, the issue of kinematic reference frame and rotations to describe motion has not been standardized.

The difficulty of converting between the multiple existing mathematical definitions of patellar kinematics or even understanding them properly is one of the major challenges for answering these questions. When we consider rotations, the way they are described has many implications. Researchers should be aware of these implications when they work with kinematic data concerning the patello-femoral joint. The choice of coordinate systems and the mathematical definition for describing patello-femoral kinematics can lead to substantial differences in the resulting curves and how one interprets whether normal kinematics have been established. Therefore, studies without a detailed description of the underlying definitions [8,9,10,11,12] are of limited value for researchers and clinicians.

There is a recommendation on definitions of joint coordinate systems for various joints from the International Society of Biomechanics. However, they do not provide a recommended definition for the knee joint, especially not for the patello-femoral joint [13,14,15]. However, since the publication of these recommendations, a lot of research has been conducted for the patello-femoral joint kinematics. To describe these kinematics, a definition using a floating axis for patellar spin [16], which has been recommended by Bull et al. [17], has been increasingly used in the past few years. This definition follows the same principle used by Grood and Suntay [18] for the tibio-femoral joint.

Nevertheless, there are still lots of studies that use more uncommon conventions for patello-femoral rotations which can lead to fundamental changes in the values for patellar flexion, spin and tilt [6,17]. Therefore, for interpretation of patello-femoral kinematics data a clear understanding of the underlying conventions is needed. If data with different underlying definitions should be compared, the ability to transform between several conventions is very helpful, but rarely described in literature.

The aim of this article is to give an overview of the different methods that can be used to describe patellar kinematics, with a particular focus on rotations. In contrast to previous publications, this article will consider the implications associated with each definition in greater detail. Additionally, the mathematics behind the patellar rotations will be given, including ways to convert data between some of the most common definitions. Therefore, the purpose is to enable researchers to choose the definition of patellar kinematics that is most suitable to them, as well as allow them to easily perform any conversions necessary to be able to compare the outcomes of different studies. As Supplementary Material, a Matlab template is provided to perform the most common conversions easily.

## 2. Materials and Methods

### 2.1. Coordinates and Definitions

**Figure 1:**
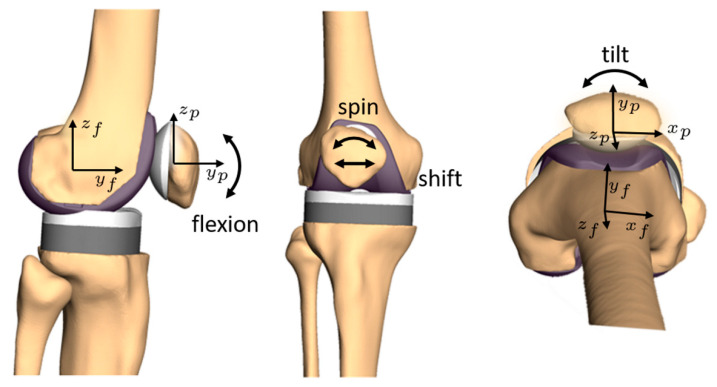
Overview on patellar kinematics and underlying coordinate systems. (**Left**): patellar flexion in a sagittal view, (**center**): medio-lateral movement (shift) and patellar spin in an anterior view, (**right**): patellar tilt in a proximal view. The exact location and orientation of the rotation axes depend on the chosen definition and is therefore not shown in detail. The patellar axes are labelled with x_p_, y_p_, z_p_ and the femoral axes with x_f_, y_f_, z_f_.

In the following description, two reference frames will mainly be used: one attached to the femur and another to the patella, with the directions x, y and z pointing laterally, anteriorly and proximally, respectively, in full extension. If the femur or patella rotate and translate during flexion, the attached coordinate systems will follow the bones’ movements. Figure 1 shows a possible system of axes for the patellar kinematics. Since the exact orientation depends on the chosen definition, it is neither described nor shown in more detail. The question of how these directions can be identified, i.e., which landmarks can be used, is highly dependent on the data and the measurement methods of every single study and will therefore not be deeply discussed. A possibility to define a reference frame for the patella is presented and validated by Innocenti et al. [19].

The most clinically relevant kinematic parameters are patellar shift, patellar flexion (α), spin (β) and tilt (γ) [5,17,20,21,22]. The patella’s resulting movements are relative to the femur as follows: patellar flexion is defined by the relative rotation of the patella around the medio-lateral flexion axis (*x*-axis). In most cases, the *femoral* flexion axis is used as the rotation axis for patellar flexion. In a clinical context, patellar spin and tilt are represented by the rotations around the local *patellar* y- and z-axes, respectively. Alternatively, it is also common to use a floating axis for patellar tilt [23,24] or patellar spin [25,26,27]. The patellar shift is the translation of the patella in the medio-lateral direction with respect to the femur or the trochlear groove. Usually, the patellar shift is given as the patellar movement in the direction of the *femoral x*-axis, which can be calculated by projecting the vector of the relative patellar translations on the normalized direction of the femoral flexion axis. The values of shift, flexion, spin and tilt for a given instance of patellar motion can differ considerably from this intuitive understanding if different definitions are used. Figure 1 shows the described components of the patellar kinematics schematically without giving precise axes and signs because these are dependent on the chosen definition.

The movements of the patella in the sagittal plane are highly dependent on the definition of the origin of the femoral coordinate system. If individual anatomical landmarks are used to define this origin, the comparison between different subjects can be problematic. However, since these movements are of secondary clinical interest [17] and are controlled mainly by joint geometry [28], they are not discussed here.

### 2.2. How Rotations Can Be Described

There are several ways to describe the rotational state of an object in a coordinate system. For example, well-known methods include rotational matrices, quaternions, projected angles, helical axes, and extrinsic and intrinsic rotation sequences. In biomechanics, representations allowing the comparison of rotations around the individual joint axes are needed. Therefore, rotation matrices, quaternions and helical axes are only suitable to process data, but not to interpret the results of studies according to patellar kinematics.

First, a fundamental property of rotations should be noted: rotations are not commutative. This means that—regardless of the mathematical description—the resulting pose for a sequence of rotations about multiple axes is generally not equal to the same rotations in another sequence. This statement remains valid for both multiple rotations in time and rotation sequences to describe a pose at one point in time.

To describe any rotation in three-dimensional (3D) space, a rotation sequence consisting of three consecutive rotations around three axes can be used. If the orientation of the axes is changed by each elemental rotation, the sequence is referred to as *intrinsic*. For initial coordinate axes ξ, η, ζ, the axes after the first rotation are written as ξ’, η’, ζ’ and after the second rotation as ξ’’, η’’, ζ’’. If all rotations occur around the axes as they are in their initial orientation, independent of previous rotation steps, the rotation sequence is called an *extrinsic* rotation.

**Figure 2:**
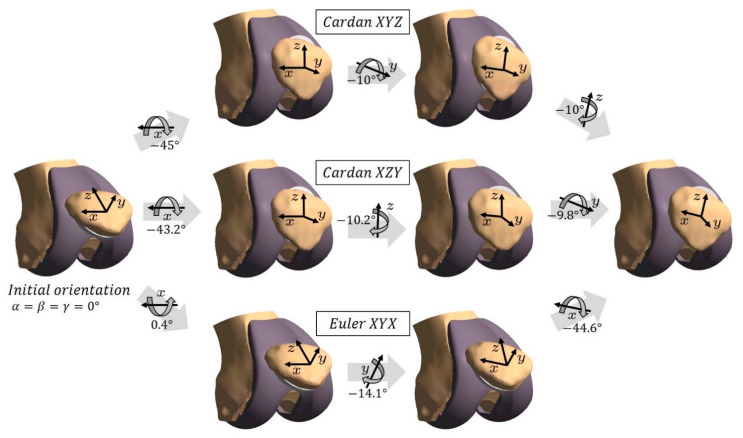
Illustration of three exemplary rotation sequences representing one particular patellar orientation. All sequences start in a patellar orientation where the patellar axes are parallel to the femoral axes. The upper and middle paths show the Cardan sequences XYZ and XZY, respectively, and the lower path represents the Euler sequence XYX.

In biomechanical literature, sometimes an intrinsic sequence of rotations around *three different axes* is called an Euler rotation, and the associated angles are termed *Euler angles* [29,30]. The correct term for these angles is in fact *Cardan angles* or *Tait-Bryan-angles*. Actual Euler angles are an intrinsic rotation sequence, which uses the same axis for the first and the third rotation (e.g., rotations around the axes ξ, η’, ξ’’) [31]. While Cardan angles can be used for a biomechanical description of patello-femoral rotations, Euler angles are difficult to relate to the clinical terms of patellar flexion, spin and tilt. Figure 2 shows three different rotation sequences which lead to a particular patellar orientation.

Two disadvantages are occasionally associated in connection with Cardan angles. The first, sequence dependency, can simply be solved by appropriate standardization of the sequence [32]. The second is termed gimbal lock, which occurs when the second angle of the sequence is ±90° [33]. In this case, the axes of the first and third rotation are collinear, and one degree of freedom is lost. Since the patellar spin and tilt do not reach absolute values of 90° [6,7] sequences with either of these in the middle position are free from this problem for the description of patellar kinematics.

**Figure 3:**
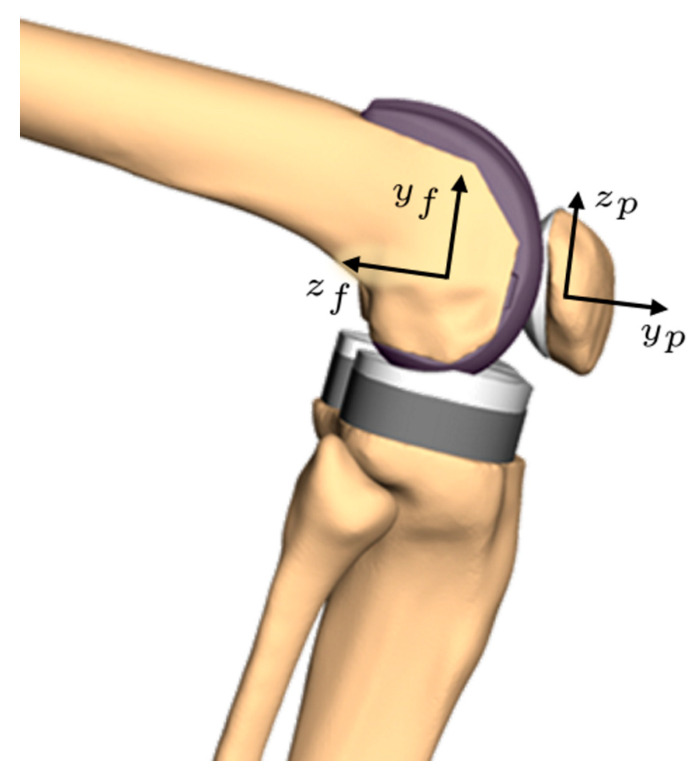
Illustration of the switch of meaning of patellar spin and tilt for an absolute patellar flexion angle of 90°, if described with respect to the femoral coordinate system. Rotating the patella around the anatomical patellar proximo-distal axis (z_p_) equals a rotation around the femoral anterior-posterior axis (y_f_) for 90° of patello-femoral flexion. The z_f_- and y_p_-axes show a similar pattern.

If femoral axes are used to describe the patello-femoral rotations [34], the interpretation of the rotations around the femoral anterior-posterior and proximo-distal axes as patellar spin and patellar tilt, respectively, are in general no longer correct. For a patellar flexion near 0° the deviation remains small, but for one of 90° the femoral tilt axis is parallel to the patellar spin axis in a sagittal view. The same applies to the femoral rotation axis and the patellar tilt axis. In this case, the calculated values for tilt and spin of the patella are switched, in addition to a possible inversion of their sign, relative to the common clinical interpretation [17] (see also Figure 3). Researchers should be aware that Cardan sequences ending with patellar flexion (ZYX and YZX) show the same pattern (e.g., [35]).

For knee kinematics, it seems that there exists another valuable way to describe the rotations. It is called the *three-cylinder open-chain representation*. For the tibio-femoral joint, it was first mentioned by Grood and Suntay in 1983 [18]. It was suggested in 2009 by Merican and Amis [36] and later on proven in 2014 by MacWilliams and Davis [37] that the Grood and Suntay definition is equivalent to a Cardan XYZ-sequence. This sequence was regardless found suitable to describe the tibio-femoral kinematics [38,39].

For the patello-femoral joint, the three-cylinder open-chain representation was first applied in 1992 by Hefzy et al. [16]. Since this way to describe patellar kinematics was recommended in a method paper [17], it is frequently used. The proof of the equivalence of the Grood and Suntay rotations and a Cardan sequence for the tibio-femoral joint [37] can be carried out analogously for the patello-femoral joint. Therefore, the three-cylinder open-chain representation of the patello-femoral kinematics is—from the rotations point of view—equivalent to the use of the Cardan sequence XYZ. Since Bull et al. [17] do not describe translations in a sagittal plane, their recommendation is to measure the patellar shift relative to the femoral medio-lateral axis and describe the patellar rotations with respect to the femoral axes as a Cardan XYZ-sequence.

The methods, which use a sequence of rotations around rotated or not rotated coordinate axes, lead to the inherent possibility to receive a description of the pose of the associated body very straightforward by executing the rotations one after the other. Nevertheless, some authors [40,41] determine the rotations of the patella by projecting the patellar coordinate vectors on the planes of the femoral coordinate system and calculate the angle between these projected vectors and the associated vectors from the femoral system. For instance, the patellar flexion can be calculated by projecting the y-vector of the patella onto the sagittal plane of the femoral coordinate system and calculating the angle between this projection and the y-vector of the femoral system. If a projection is carried out onto the patellar coordinate planes, a rotation relative to the patellar axes can be calculated. This method in general does not lead to values for patellar flexion, spin and tilt which can be executed in sequence to acquire the full rotation of the patella relative to the femur. For every projection plane, there are two vectors that can be projected to calculate the rotation around the axis perpendicular to this plane. As the results of this paper will show, the calculated angles for these two projected directions differ a lot for 3D rotations. For example, the calculation of the patellar flexion as the angle between the patellar *y*-axis projected onto the sagittal plane of the femoral system differs from the same calculation with the z-axes. Therefore, the angles are highly dependent on the choice of planes and axes.

### 2.3. Conversions

**Figure 4:**
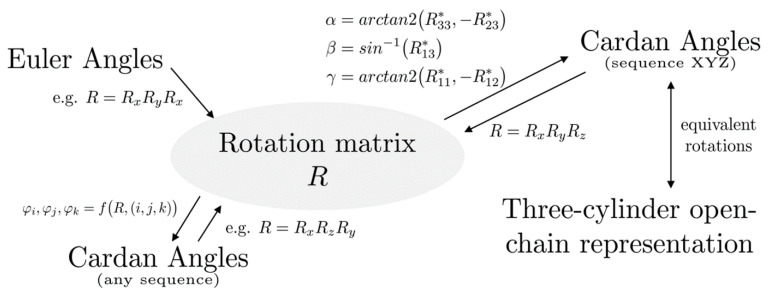
Overview of transformation paths between different descriptions of patellar rotations.

A method to convert a rotation from one definition to another is to calculate the rotation matrix representing the rotation and use this matrix to calculate the parameters of the desired definition. To that end, methods will be given to convert the common definitions into matrix form and back. Figure 4 shows an overview of the conversion paths introduced in this paper. The conversions are first given for intrinsic and extrinsic rotation sequences.

#### 2.3.1. Rotation Sequences

With the standard uniaxial 3D rotation matrices R_x_(α), R_y_(β) and R_z_(γ) for the rotations around x, y and z, the rotation matrix R of the full 3D rotation can be calculated by matrix multiplication. For an extrinsic rotation sequence, the resulting matrix R is the matrix product of the corresponding uniaxial rotation matrices, expressed in the opposite sequence order from left to right. For an intrinsic rotation sequence, the order of the uniaxial rotation matrices is inverted. For example, the rotation matrix R = R_x_(α)∙R_y_(β)∙R_z_(γ) represents the extrinsic rotation sequence ZYX and the intrinsic sequence XYZ. It is important to note that this method works the same for Euler angles.

The formulas for calculating the angles α, β and γ for a specific sequence from a given rotation matrix R* with the entry R_st_* in line *s* and column *t* are obviously dependent on the sequence. Rotating around a particular axis twice within one sequence leads to angles that are hard to interpret clinically. Only the equations for Cardan angles are therefore given here.

For certain sequences, the following method is often shown in literature (e.g., intrinsic XYZ-sequence [32,42]). Here, a general formulation which can be used for any sequence, will be introduced.

Let (i,j,k) be a tuple of three different indices with (i,j,k) ∈ {1,2,3}. Then every (i,j,k)-tuple represents a Cardan sequence (e.g., (i,j,k) = (1,3,2) stands for the sequence XZY). To calculate the angles of the patello-femoral rotations, the sign sgn_ijk_ of a sequence tuple is needed. The sign is equal to 1 if the tuple (i,j,k) can be created from the tuple (1,2,3) by an even number of transpositions. If the number of transpositions is odd, sgn_ijk_ is equal to −1 [43]. The equations

φ_i_ = arctan2 (R_kk_*, −sgn_ijk_∙R_jk_*) (1)

φ_j_ = sin^−1^ (sgn_ijk_∙R_ik_*) and (2)

φ_k_ = arctan2 (R_ii_*, −sgn_ijk_∙R_ij_*) (3)

directly lead to the sought angles for flexion (α = φ1), spin (β = φ_2_) and tilt (γ = φ_3_). The proof for Equations (1)–(3) is given in the Supplementary Materials.

#### 2.3.2. Projected Angles

For the reconstruction of the 3D orientation from projected angles, giving a closed description of all 64 possible axis-plane projection combinations is not feasible in this article. Only a brief overview of the derivation of the equations will be given here. Every given projected angle can be used to determine a semicircle containing all the unit vectors that would lead to this angle if projected. If the j-th axis is projected onto the femoral (or patellar) coordinate plane, the parametrized semicircle can be set to equal the j-th column (or row, in the case of the patellar plane) vector of the rotation matrix. The orthogonality of rotation matrices (pairwise orthogonality of rows and columns, Euclidian norm of every row and column equals 1, third row/column is cross product of first and second row/column) gives the conditions to determine the parameters and the missing entries of R.

#### 2.3.3. Helical Axes

Helical axes are a way to represent a 3D-movement using only one rotation axis for every time step. The movement is described as a translation along this axis and a rotation around it. This method is sometimes used to analyze the actual joint rotation axis, especially for the tibio-femoral joint [44,45]. This description has also been sometimes used for the patello-femoral joint [46,47]. Consequently, the ideas for the conversion of the rotations are discussed here briefly.

The direction of the rotation axis is given by the helical axis. Thus, the Rodrigues’ rotation formula [48] can be used to rotate a vector around any given rotation axis. Matrix representation of a helical axis rotation can be achieved by applying this formula to the unit vectors. The rotated unit vectors give the columns of the associated rotation matrix. For the opposite conversion, the direction of this axis is the eigenvector to the eigenvalue of 1, while the angle can be obtained from the other eigenvalues [49,50]. For more details [51] is recommended.

#### 2.3.4. Three-Cylinder Open-Chain Representation

As stated previously, the angles in the three-cylinder open-chain representation [16,17] are equivalent to the angles of the intrinsic XYZ rotation sequence. Therefore, the methods used for this sequence can also be applied to convert rotations from or into this convention.

### 2.4. Data Processing and Validation

To compare the definitions described earlier in this paper, kinematic data from a previously published and validated musculoskeletal model of the lower right extremity simulating a squat motion [20] was used. This simulation model was implemented in the software SIMPACK (V9.7, Dassault Systèmes Deutschland GmbH, Gilching, Germany). The rotations of the patella relative to the femur were evaluated for a squat motion in all possible Cardan sequences directly in the software. These were used as reference data for verifying the implementation of the previous formulas in MATLAB (R2018a, Mathworks, Natick, Massachusetts, USA). The deviations between the curves were quantified by calculating the Root Mean Squared Error (RMSE).

## 3. Results

The dataset for a squat includes tibio-femoral flexion angles from 0° to 90°. The discrepancy between the angles according to the different definitions increases with the tibio-femoral flexion angles. Based on the data for the Cardan XYZ-sequence, the angles for the five remaining Cardan sequences and the projected angles were calculated. In order to validate the conversion process, the Cardan sequences for the same dataset were evaluated directly from the general multibody software SIMPACK as reference. The deviation of these results to the values from converting the XYZ data do not exceed 0.0001° (RMSE < (6.13 × 10^−5^)° for all sequences and angles).

### 3.1. Cardan Angles

**Figure 5:**
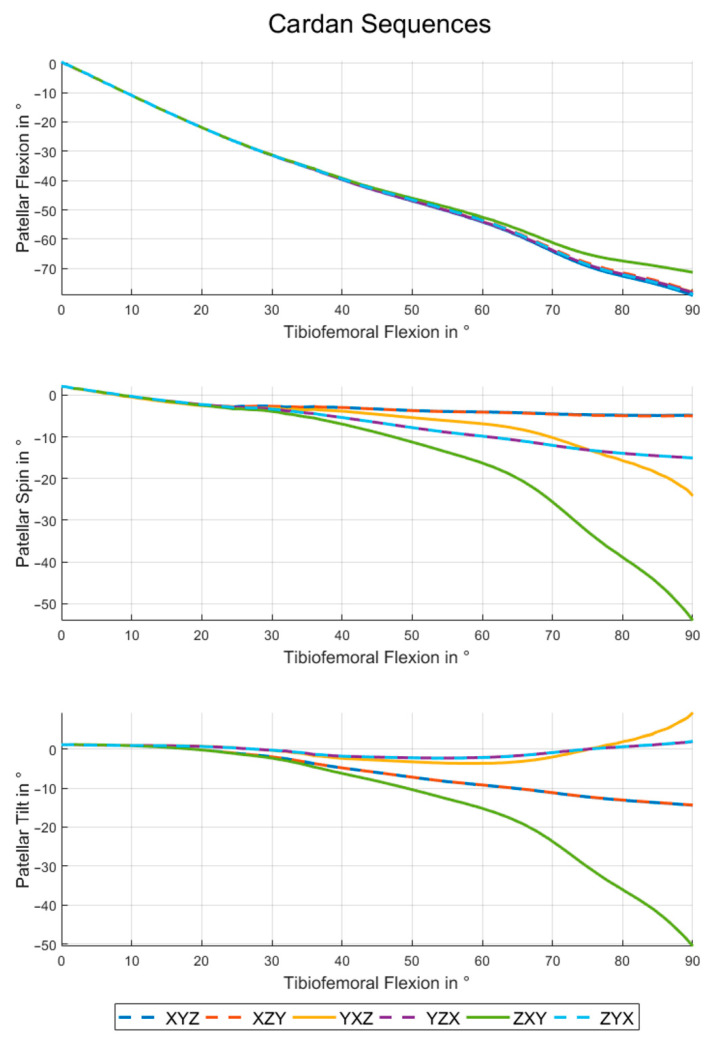
Patellar kinematics for the Cardan sequences. Patellar flexion (**top**), spin (**center**) and tilt (**below**) during a squat motion for all Cardan sequences.

Figure 5 shows the rotation angles for all possible Cardan sequences. The choice of definition has a minor effect on the patellar flexion values. All Cardan sequences except for ZXY have very similar flexion angles. The maximum RMSE within this group is 0.79° (see also Table 1). The patellar flexion angle of the ZXY-sequence differs up to 7.83° from the others (RMSE ≥ 3.71°).

In terms of patellar spin and patellar tilt the Cardan XYZ- and XZY-sequences are very close (spin: RMSE = 0.09° and tilt: RMSE = 0.03°) as Table 2 and Table 3 show. The same is true for YZX and ZYX (spin: RMSE = 0.004° and tilt: RMSE = 0.03°). The sequence ZXY leads to curves which differ considerably from those of other sequences (RMSE up to 27.72°). These deviations are particularly large and grow increasingly for higher tibio-femoral flexion angles, and reach a maximum of 49.22° for the patellar spin and 59.91° for the patellar tilt.

### 3.2. Projected Angles

**Figure 6:**
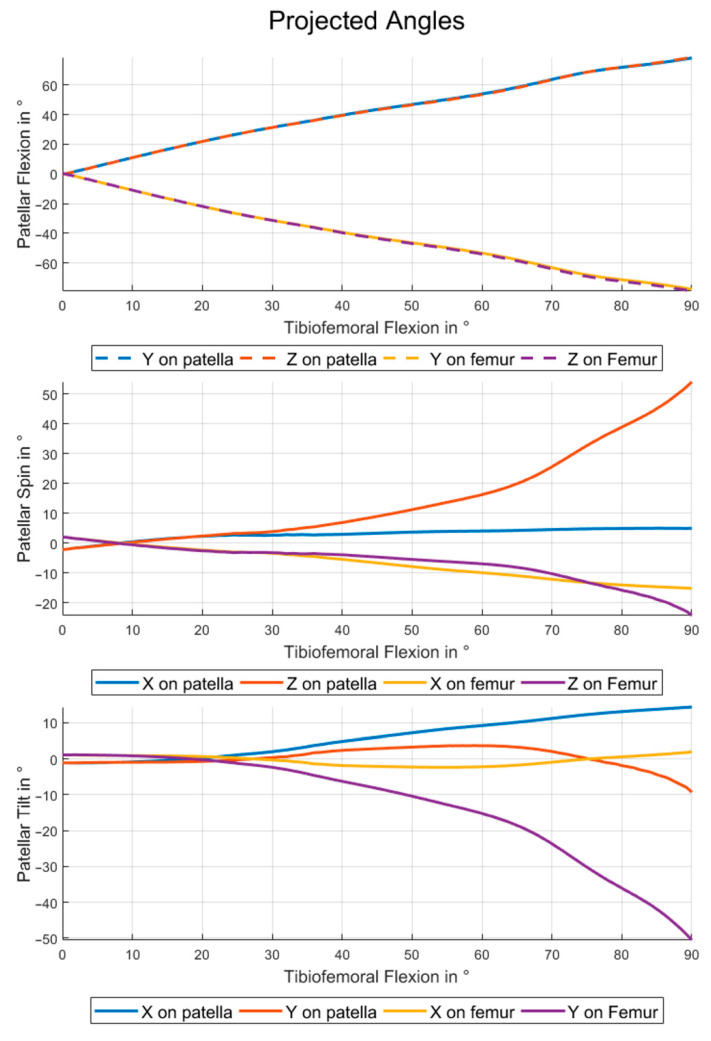
Patellar kinematics using projected angles. Patellar flexion (**top**), spin (**center**) and tilt (**below**) during a squat motion for all possible ways to calculate the angles by projection.

The projected angles can be divided into two groups. Projecting the patellar reference frame vectors onto the femoral planes gives patello-femoral rotations with respect to the femoral system. Therefore, the angles for patellar flexion are close to the negative of the angles obtained by projecting onto the patellar planes (see also Figure 6, top). The flexion angles calculated by projecting the *y*- (or *z*-) axis on the femoral standard plane are equal to the patellar flexion angles from the Cardan XZY- (or XYZ- for the *z*-axis) sequence as the RMSE of 0° for these combinations shows in Table 1. The same pattern is visible for the patellar spin and tilt if the projections on femoral planes and Cardan sequences starting with y- and z-axes are analyzed (see also Table 2 and Table 3).

## 4. Discussion

The first objective of this study was to show the considerable impact of different mathematical definitions on how patello-femoral kinematics are conveyed. A framework was thereby developed to enable researchers to choose the definitions for the patellar kinematics which best fit their needs and to convert patellar kinematics between the different conventions. In this way, comparability and interchangeability between different studies may be facilitated.

The results obtained from the conversions we have presented using the described formulas are in excellent agreement with those obtained from the evaluation using a general multibody software package (RMSE < (6.13 × 10^−5^)° for all sequences and angles). Therefore, the derived equations and their implementation were verified.

The presented results indicate clearly that the chosen definition for patello-femoral angles has an impact on the description of patello-femoral kinematics. The curves for patellar flexion, spin and tilt deviate not only in magnitude but also in their characteristics (see Figure 5 and Figure 6). Therefore a correct interpretation of patello-femoral kinematic data seems to be impossible if the details of the convention used are not clear.

Deviations between the results with different definitions only occur if rotations around at least two standard directions differ from zero. This condition is based on the fact that the differences are caused by changes in rotation axes for subsequent rotations. For increasing tibio-femoral flexion angles the absolute patellar flexion angle reaches values close to 80°, while the absolute values of patellar spin and tilt stay relatively small if the rotations are considered around the patellar axes. Therefore, the rotation around the *x*-axis and its position in the Cardan sequence has a special impact on the data for high tibio-femoral and patello-femoral flexion angles. In the case of small patellar spin and tilt, it can be assumed that the rotation axes that occur before the patellar flexion in the Cardan sequence almost agree with the associated femoral coordinate system axes. The rotations placed after the flexion in the sequence can be interpreted as rotations around associated patellar body axes. For the first and last rotations of a Cardan sequence these statements are exactly true.

For the patello-femoral kinematics this means that the first rotation of a sequence is always around the associated axis of the femoral coordinate system and the last rotation is around a patella-fixed axis, as previously shown in Figure 2. The floating axis between these two is close to the associated patellar axis if the sequence starts with the patellar flexion and is close to the associated femoral axis otherwise. In accordance with the previous explanations the two Cardan sequences starting and ending with the patellar flexion (XYZ/XZY and YZX/ZYX) show very small differences (see Figure 5, and Table 1, Table 2 and Table 3).

For 90° of absolute patellar flexion angle patellar spin and tilt would swap its values if the patellar flexion would be changed from the first position (i.e., tilt and spin close to patellar body fixed rotations) to the third position (i.e., tilt and spin close to femoral body fixed rotation). This is caused by the inherent change from patellar to femoral coordinate system. For the squat cycle showed here, only 80° of absolute patellar flexion are reached. Nevertheless, the described effect can be seen, if the maximum patellar tilt for the sequences XYZ and XZY (15.0°) is compared to the patellar spin for YZX and ZYX (15.7°).

One of the main difficulties in using Cardan sequences to describe rotational poses is the gimbal lock, which occurs if the second rotation of the sequence is equal to 90°. In this case, the axis of the third rotation is parallel to the direction of the first one (or its negative) and the rotation loses one degree of freedom. The absolute values for spin and tilt are far from 90° for healthy knees [6,7]. Therefore, this situation only exists if the patellar flexion is the second rotation of the sequence. Even if the maximum absolute values for patellar flexion in the shown results are less than 80°, an increase of spin and tilt can be mentioned for the YXZ sequence and a comparably larger one for the ZXY sequence. (see Table 2 and Table 3). The reason why ZXY differs more from the sequences without the x-rotation on second position than YXZ is that the patello-femoral rotation around the femoral *z*-axis is bigger than around the femoral *y*-axis. These effects are making the interpretation of the patellar kinematics in the ZXY and YXZ sequence quite laborious and this description differs a lot from a clinical understanding for spin and tilt. Therefore these sequences are not recommended.

The projected angles also show big differences dependent on the choice of which axis is projected onto which coordinate system. Figure 6 indicates clearly that this method is very sensitive to the choice of projection axis and that this effect increases with growing angles in the other two dimensions. The flexion angles are robust against switching from *y*- to *z*-axis for projection, while comparable changes for spin and tilt cause completely different curves due to the relatively higher patellar flexion angles.

For small angles of spin and tilt the projected angles for flexion on the patellar (or femoral) planes can be interpreted as flexion with respect to the patellar (or femoral) system. Due to the high flexion angles, the same interpretation cannot be applied to spin and tilt. For spin and tilt the values are also highly depending on the choice of axis for projection.

It was shown that the deviations between the curves for patellar flexion, spin and tilt for the different kinematic definitions can completely change in both, magnitude and characteristics of the curves. Nevertheless, the differences are not big enough in every case that inadvertently comparing results based on different definitions would be obvious at first glance. Therefore the problem explored by this study should always be considered when dealing with patellar kinematics.

Patellar maltracking is known as possible cause of anterior knee pain [4,5,52,53,54,55], but it still remains unknown, how critical patellar kinematics can be distinguished from others [6]. To increase the biomechanical knowledge about how anterior knee pain can be prevented or treated, it will help to bring the available data from the literature together. Therefore, it is essential to properly understand the underlying definitions of every single study and to carefully transform the data into one representation, that can be compared across the available literature. For most of the studies available in literature the given transformations from this study can be utilized.

If researchers are free to choose a convention for their own study, the use of Cardan sequences is recommended due to the available straight forward methods for calculation, conversion and interpretation. Even if all Cardan sequences are theoretically suitable to represent the patello-femoral kinematics in a correct way, some of them are better for intuitive interpretation from a biomechanical side of view. The definitions which are closest to the clinical understanding of patellar flexion, tilt and spin are the two Cardan sequences beginning with patellar flexion (XYZ and XZY), where patellar flexion is given around the *femoral* flexion axis. The last rotation of the sequence is given exactly around the associated *patellar* axis while the second rotation of the sequence is executed around the floating axis perpendicular to the other two. Therefore, the rotation (out of spin and tilt) with the most importance for a certain study should be chosen as last rotation of the sequence. The higher clinical relevance of tilt compared to patellar spin will qualify the Cardan sequence XYZ as recommendation for most studies.

## 5. Conclusions

In this study the most common definitions for patello-femoral rotations and the most important conversions between them were described. Using kinematic data of a validated squat motion based on motion capture, it was shown that the angles describing the patellar kinematics are highly dependent on the underlying convention.

If researchers are not aware of the described deviations, misinterpretation of results is very likely, which is critical for clinically relevant studies. Additionally, the comparison of different studies should only be executed if equivalence of the used conventions can be ensured or if the data are carefully transformed. Therefore, the methods from this study will help to uncover the complex relationship between patellar kinematics and anterior knee pain.

## Figures and Tables

**Table 1:** RMSE of patellar flexion curves for Cardan sequences and projected angles in °.

	XYZ	XZY	YXZ	YZX	ZXY	ZYX	Y on Patella	Z on Patella	Y on Femur	Z on Femur
XYZ	0	-	-	-	-	-	-	-	-	-
XZY	0.79	0	-	-	-	-	-	-	-	-
YXZ	0.46	0.36	0	-	-	-	-	-	-	-
YZX	0.43	0.4	0.05	0	-	-	-	-	-	-
ZXY	3.71	2.96	3.25	3.28	0	-	-	-	-	-
ZYX	0.39	0.44	0.26	0.25	3.4	0	-	-	-	-
Y on patella	111.48	110.71	111.06	111.09	108.11	111.1	0	-	-	-
Z on patella	111.49	110.72	111.07	111.1	108.12	111.12	0.25	0	-	-
Y on femur	0.79	0	0.36	0.4	2.96	0.44	110.71	110.72	0	-
Z on femur	0	0.79	0.46	0.43	3.71	0.39	111.48	111.49	0.79	0

**Table 2:** RMSE of patellar spin curves for Cardan sequences and projected angles in °.

	XYZ	XZY	YXZ	YZX	ZXY	ZYX	X on Patella	Z on Patella	X on Femur	Z on Femur
XYZ	0	-	-	-	-	-	-	-	-	-
XZY	0.09	0	-	-	-	-	-	-	-	-
YXZ	8.27	8.18	0	-	-	-	-	-	-	-
YZX	6.56	6.48	3.09	0	-	-	-	-	-	-
ZXY	24.37	24.28	16.19	18.06	0	-	-	-	-	-
ZYX	6.56	6.48	3.09	0.004	18.06	0	-	-	-	-
X on patella	7.85	7.93	15.47	14.24	31.53	14.24	0	-	-	-
Z on patella	31.44	31.53	39.56	37.94	55.72	37.94	24.28	0	-	-
X on femur	6.56	6.48	3.09	0	18.06	0.004	14.24	37.94	0	-
Z on femur	8.27	8.18	0	3.09	16.19	3.09	15.47	39.56	3.09	0

**Table 3:** RMSE of patellar tilt curves for Cardan sequences and projected angles in °.

	XYZ	XZY	YXZ	YZX	ZXY	ZYX	X on Patella	Y on Patella	X on Femur	Y on Femur
XYZ	0	-	-	-	-	-	-	-	-	-
XZY	0.03	0	-	-	-	-	-	-	-	-
YXZ	11.07	11.04	0	-	-	-	-	-	-	-
YZX	9.64	9.61	2.27	0	-	-	-	-	-	-
ZXY	16.69	16.72	27.72	26.14	0	-	-	-	-	-
ZYX	9.65	9.62	2.24	0.03	26.15	0	-	-	-	-
X on patella	19.36	19.33	9.38	9.92	35.39	9.91	0	-	-	-
Y on patella	9.38	9.35	6.79	4.67	24.45	4.7	11.07	0	-	-
X on femur	9.65	9.62	2.24	0.03	26.15	0	9.91	4.7	0	-
Y on femur	16.69	16.72	27.72	26.14	0	26.15	35.39	24.45	26.15	0

## Data Availability

The dataset used for this study is available as Supplementary Material (S3).

## Supplementary Materials

The following are available online at https://www.mdpi.com/article/10.3390/ma14247644/s1, S1: Matlab-template to transform patellar rotations between the described conventions, S2: Instructions for use of the template (S1), S3: Dataset used in this paper in XYZ-Cardan sequence form, S4: Proof for the conversion formulas (1)–(3).
