# Supplementary material for: The Influence of Mathematical Definitions on Patellar Kinematics Representations
Source: Materials (Basel). 2021 Dec 11;14(24):7644. doi: 10.3390/ma14247644 (PMC8709478; doi:10.3390/ma14247644)
Supplement: Supplementary file 1 [file materials-14-07644-s001.zip › S2_Instructions.pdf]

Instructions for using the template

Open the file “S1_Template_Transformations.m” in Matlab. It was developed and tested in Matlab R2018a.

The provided data sample is stored as Matlab-Matrix-File (XYZ_Sequence_Example.mat) and it contains the angles of a squat in XYZ-sequence. The first column of this file contains the flexion angles.

1. Preparations

Put your data into a Matlab-Data-File (.mat), as shown in figure 1. Use the following structure:

First column: Time or tibiofemoral flexion angle (only used as x-axis for charts)

Columns 2-4:

If your input is a Cardan sequence:

Column 2	Column 3	Column 4
rotation around x	rotation around y	rotation around z

If your input is an Euler sequence (first and last rotation around same local axis):

Column 2	Column 3	Column 4
First rotation	second rotation	third rotation

SquatExample

145x4 double

	1	2	3	4	5
1	-0.3530	0.8314	2.1566	1.0967	
2	0.1358	0.2282	2.0703	1.1037	
3	0.6585	-0.4231	1.9672	1.1076	
4	1.2153	-0.9412	1.7652	1.1293	
5	1.8077	-1.6081	1.5587	1.1414	
6	2.4369	-2.3382	1.4648	1.1256	
7	3.1034	-3.0926	1.3073	1.1051	
8	3.8075	-3.7793	1.0902	1.0983	
9	4.5513	-4.7797	0.8756	1.0828	
10	5.3370	-5.5923	0.7071	1.0476	
11	6.1655	-6.6469	0.4378	1.0290	
12	7.0381	-7.4983	0.2705	0.9867	
13	7.9539	-8.6729	0.0091	0.9492	

Figure 1: Example for input data

2. Execution of the calculations

a. Start the execution of the script by pressing the “run”-button in your Matlab window.

b. Choose your input data file (see also figure 2).

c. Choose the further options (see also figure 3).

- Choose <u>one</u> input definition from the left list, according to your input data.
- Choose one or several definitions for outputs from the right list (use ctrl to select multiple output types)
    - i. If you choose “projected angles” as output, you will get all options to choose, which axis should be projected on which coordinate system. Here also multiple options can be selected (see also figure 4)
- Choose the datatype for the x-axis from the options on the right of the cancel button.
- Press OK.

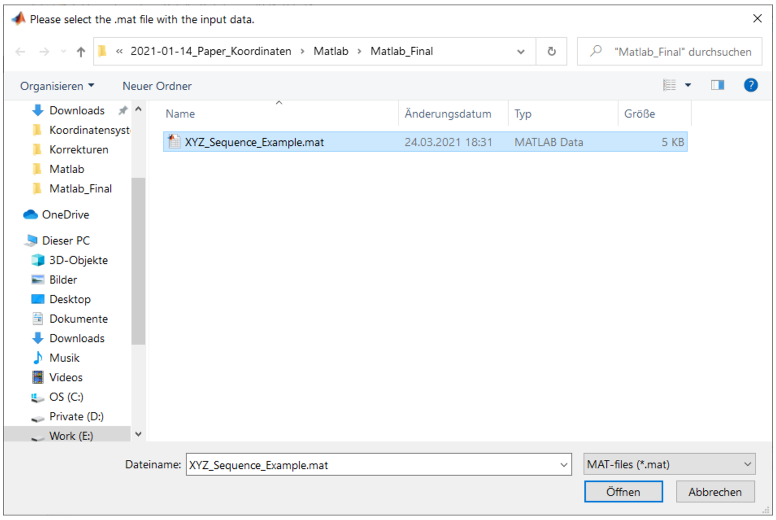

Figure 2: Choose your input file.

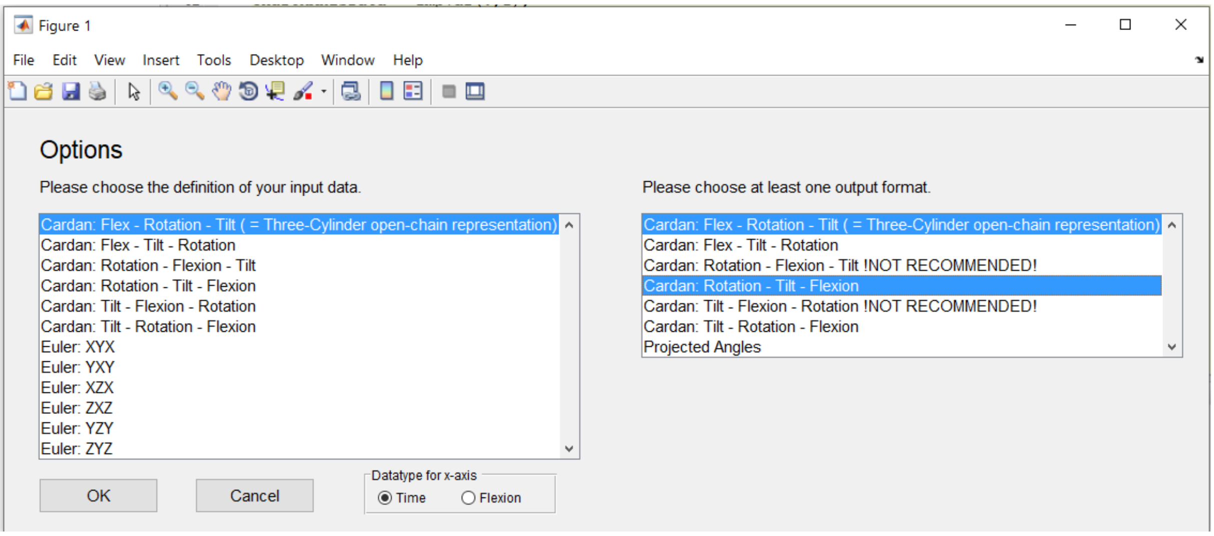

Figure 3: Window to choose the desired options.

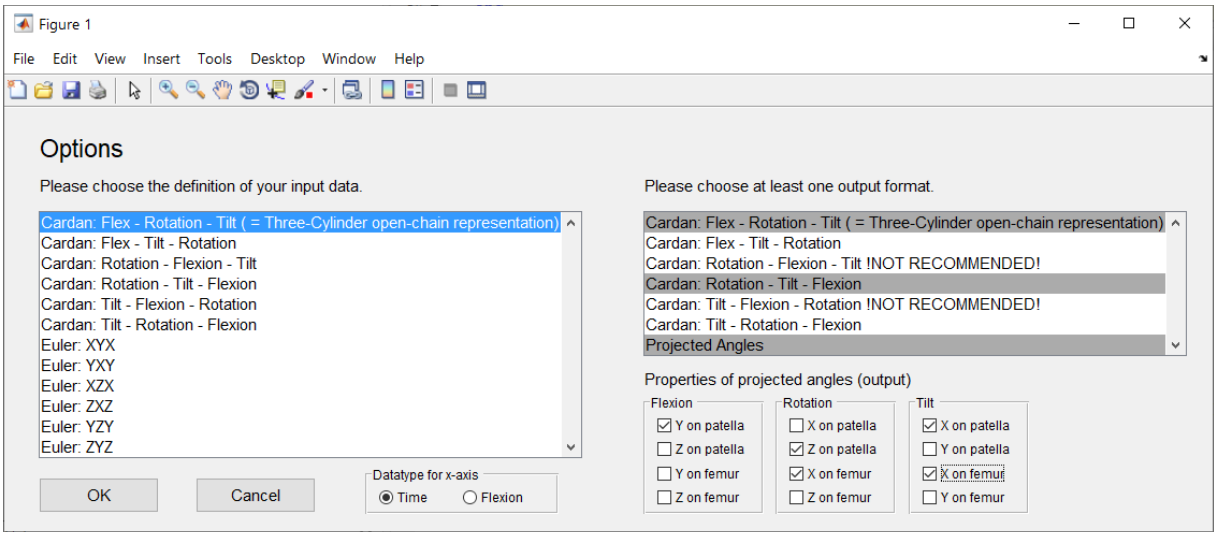

Figure 4: Options-window with visible options for the projected angles output.

3. Results

Matlab will plot the chosen outputs in a basic way and you get your results in the workspace variables (see also figure 5). For every Cardan sequence there is a separate variable with three columns (1. Flexion (x), 2. Rotation (y) and 3. Tilt (z)). For the projected angles there is the variable “ResultsProj” with 12 columns in it. The order of columns in this variable is given by the order of options for the projected angles in figure 4 column wise from top left to bottom right. The columns of projections you did not chose will remain empty in this variable.

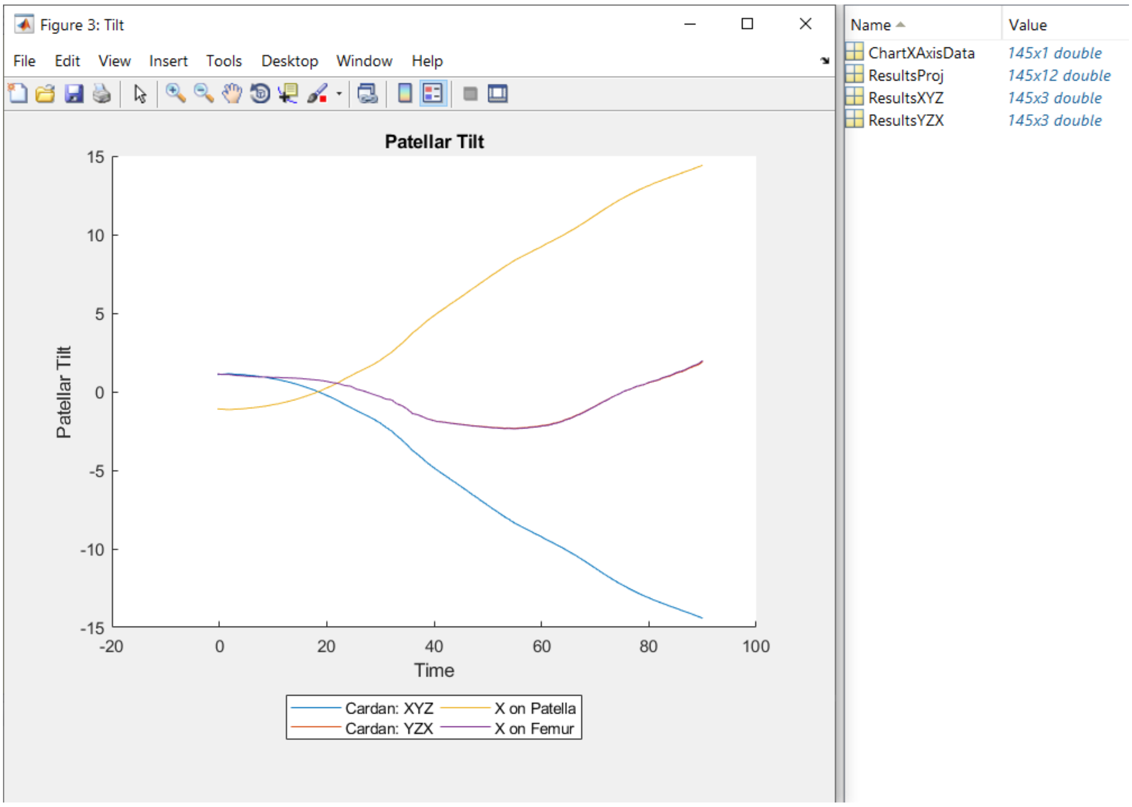

Figure 5: Example for a basic tilt-chart with some chosen outputs.
