# Supplementary material for: The Influence of Mathematical Definitions on Patellar Kinematics Representations
Source: Materials (Basel). 2021 Dec 11;14(24):7644. doi: 10.3390/ma14247644 (PMC8709478; doi:10.3390/ma14247644)
Supplement: Supplementary file 1 [file materials-14-07644-s001.zip › S4_Proof_Cardan_Conversions.pdf]

Proof for the Cardan-conversion-formulas

The formulas (1) to (3) are showed without any proof in the publication. Within this document the proof will be given for

$$\varphi_i = arctan2\big(R^*_{kk}, -sgn_{ijk}\ R^*_{jk}\big) \quad , \tag{1}$$

$$\varphi_j = sin^{-1}\big(sgn_{ijk}\ R^*_{ik}\big) \quad and \tag{2}$$

$$\varphi_k = arctan2\big(R^*_{ii}, -sgn_{ijk}\ R^*_{ij}\big) \quad . \tag{3}$$

Proof: By executing the multiplication of the uniaxial rotation matrices in every possible combination, it can be shown that the general rotation matrix R, expressed as a function of the angles, with the associated sequence tuple (i, j, k) contains the following entries

$$R_{ii} = cos\varphi_j\ cos\varphi_k \quad , \tag{4}$$

$$R_{ij} = -sgn_{ijk}\ cos\varphi_j\ sin\varphi_k \quad , \tag{5}$$

$$R_{ik} = sgn_{ijk}\ sin\varphi_j \quad , \tag{6}$$

$$R_{jk} = -sgn_{ijk}\ sin\varphi_i\ cos\varphi_j \quad \text{and} \tag{7}$$

$$R_{kk} = cos\varphi_i\ cos\varphi_j \quad . \tag{8}$$

By letting R equal the given rotation matrix R^*, it is possible to solve for the values of these angles. That means that angles φ_i, φ_j and φ_k are needed, for which equations (4) to (8) hold, given that $R(\varphi_i, \varphi_j, \varphi_k) = R^*$. Rearranging equations (7) and (8) to solve for $sin\varphi_i$ and $cos\varphi_i$ and then dividing one by the other leads to

$$tan\varphi_i = \frac{-sgn_{ijk}\ R_{jk}}{R_{kk}} = \frac{-sgn_{ijk}\ R^*_{jk}}{R^*_{kk}} \tag{9}$$

$$\Leftrightarrow \quad \varphi_i \ = arctan2\big(R^*_{kk}, -sgn_{ijk}\ R^*_{jk}\big) \tag{10}$$

which is exactly equation (1).

Equation (3) follows analogously from (4) and (5).

Equation (2) is equivalent to equation (6) after replacing R by R^*. □
